# Supplementary material for: Seminoma and Embryonal Carcinoma Footprints Identified by Analysis of Integrated Genome-Wide Epigenetic and Expression Profiles of Germ Cell Cancer Cell Lines
Source: PLoS One. 2014 Jun 2;9(6):e98330. doi: 10.1371/journal.pone.0098330 (PMC4041891; doi:10.1371/journal.pone.0098330)
Supplement: File S1 — ZIP file containing DMRforPairs output for significant regions. Please start from the html files. (ZIP) [file pone.0098330.s008.zip › figures/13666.pdf]

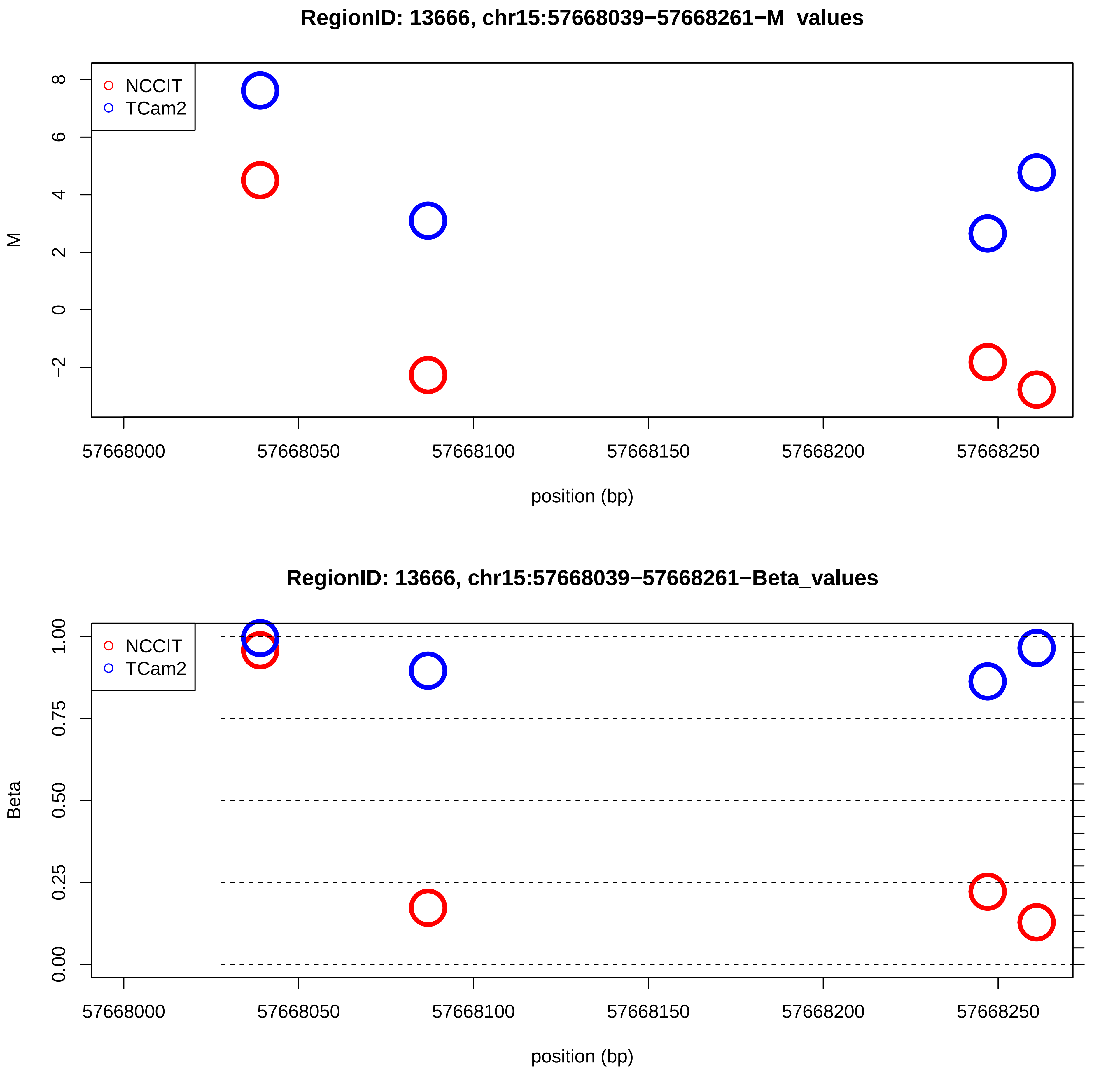
RegionID: 13666, chr15:57668039−57668261−M_values
M
position (bp)
NCCIT
TCam2
8
6
4
2
0
−2
57668000
57668050
57668100
57668150
57668200
57668250
RegionID: 13666, chr15:57668039−57668261−Beta_values
Beta
1.00
0.75
0.50
0.25
0.00
